# Hepatitis B vs. hepatitis C infection on viral hepatitis-associated hepatocellular carcinoma

**DOI:** 10.1186/1471-230X-12-64

**Published:** 2012-06-08

**Authors:** Spiros P Hiotis, Nuh N Rahbari, Gerald A Villanueva, Eunjie Klegar, Wei Luan, Qin Wang, Herman T Yee

**Affiliations:** 1 Department of Surgery, Mount Sinai School of Medicine, New York, USA; 2 Department of Surgery, University of Heidelberg, Heidelberg, Germany; 3 Department of Gastroenterology, Bellevue Hospital / NYU, New York, USA; 4 Department of Pathology, Bellevue Hospital / NYU, New York, USA; 5 Division of Surgical Oncology, Mount Sinai School of Medicine, One Gustave L Levy Place, Box 1259, New York, NY, 10029, USA

**Keywords:** Hepatocellular carcinoma, Hepatitis B, Hepatitis C, Viral hepatitis

## Abstract

**Background:**

To determine clinical-pathologic variables in patients with a new diagnosis of hepatocellular carcinoma (HCC) and underlying hepatitis B vs. C infection.

**Methods:**

Patients presenting to a single urban hospital with a new diagnosis of HCC were entered into a clinical database. Variables including number and size of tumors, presence of metastases, serum alpha-Fetoprotein, hepatitis serologies, severity of hepatic dysfunction, and presence of cirrhosis were evaluated in 127 patients.

**Results:**

Patients with hepatitis B (HBV) were more likely to develop HCC at a younger age than patients with hepatitis C (HCV) (HBV-26% under age 40, HCV-0% under age 40; p < 0.001), with greater serum alpha-Fetoprotein production (median level: HBV-1000 ng/ml vs. HCV-37 ng/ml; p = 0.002), with larger tumors (HBV-78% >5 cm, HCV-28% >5 cm; p < 0.001), in the absence of cirrhosis (HBV-40%, HCV-0%; p < 0.001), and a decreased eligibility for curative treatment (HBV-14%, HCV-34%; p < 0.05). Conversely, patients with HCV were more likely to develop HCC in association with multiple co-morbidities, cirrhosis, and older age.

**Conclusions:**

Significant clinical-pathologic differences exist among HCC patients with underlying HBV vs. HCV. These differences impact eligibility for potentially-curative therapy and prognosis.

## Background

Hepatocellular Carcinoma (HCC) is the third leading cause of cancer related mortality worldwide [1]. The global prevalence and mortality resulting from HCC is directly related to underlying risk factors for primary liver cancer in at-risk populations. Although several chronic liver diseases are associated with HCC, Hepatitis B Virus (HBV) and Hepatitis C Virus (HCV) statistically are the most commonly implicated risk factors. Combined they are responsible for 85% of total new HCC cases worldwide; 54% occurring as a result of HBV, and 31% as a result of HCV [2,3]. HBV, which is endemic in developing geographic regions such as Eastern Asia and Sub-Saharan Africa, is responsible for up to 90% of new HCC cases in such areas [4].

Epidemiologic analyses and clinical series which lead to our understanding of the behavior and natural history of HCC do not usually subset results according the underlying viral risk factors for the disease [5]. Most survival analyses from institutions with broad experience in surgical resection, transplantation, ablation, transarterial chemoembolization, or other treatments usually combine patients, irrespective of their underlying viral hepatitis status or association to other risk factors [6,7]. Therefore variations in patterns of presentation, tumor biology, or treatment outcomes for HCC according to underlying association to viral hepatitis B or C remain unclear.

Despite histologic similarities in end-organ damage and eventual carcinomas, adequate scientific rationale supports a proposed hypothesis that distinct pathophysiologic mechanisms may be responsible for hepatocarcinogenesis due to either HBV or HCV. This is based in part on marked virologic differences seen among the 2 pathogens. Hepatitis B is a DNA virus belonging to the Hepadna virus family, which persists in the hepatocyte nucleus predominantly in the form of covalently closed circular DNA (cccDNA), the functional template for viral transcription and replication [8]. Conversely, Hepatitis C is an RNA virus belonging to the flavivirus family which replicates in the hepatocyte cytoplasm, with a completely distinct life cycle and pattern of viral replication [9,10]. Differences in natural history of resulting benign liver disease is well-described among the 2 viruses [11].

In this study, an attempt has been made to evaluate potentially significant clinical-pathologic differences in HCC’s that develop in association with chronic HBV vs. HCV. Comparisons have been made among two otherwise comparable groups according to data collected upon initial cancer diagnosis at a single, urban hospital. Demographic data and widely accepted relevant clinical-pathologic features of newly diagnosed HCC’s have been collected with the intended purpose of delineating potentially significant differences in cancers which arise in HBV vs. HCV infected patients.

## Methods

### Database enrollment

Patients were enrolled into a clinical database based upon a diagnosis of HCC at a single New York City public hospital (Bellevue Hospital Center). The database was approved by the IRB of New York University Medical Center and was in compliance with the Helsinki Declaration. The overall period of enrolment spanned the years of 1993 to 2005. Data for patients diagnosed between 1993 and 2001 was collected in a retrospective manner, while data collected between the years of 2001 and 2005 was entered prospectively. This study was exempt from IRB approval according to the institutional criteria at New York University Medical Center. Hence, informed consent was not required for this study.

Demographic data including age, sex, race, and ethnicity were collected. Several clinical-pathologic features were also recorded upon initial HCC diagnosis including the following: tumor size, tumor number, presence of synchronous distant metastases, macro-vascular invasion (tumor thrombus), viral hepatitis serologies, serum biochemistries including liver function tests, serum alpha-fetoprotein, presence and severity (according to Childs-Pugh classification) of coexisting cirrhosis, and underlying co morbidities.

In subsequent retrospective analysis of database variables, patients were stratified according to underlying risk factors for HCC. In order to facilitate a cohort comparison of HBV vs. HCV as risk factors for cancer, patients with either HBV or HCV only were included for analysis. Those co-infected both with HBV and HCV, or neither hepatitis virus were excluded from this analysis.

### Diagnosis of HCC

The diagnosis of HCC was based upon histology or cytology, when tissue was available following surgical resection or biopsy. For cases in which no tissue was available, patients were diagnosed with HCC if dynamic imaging findings (CT with intravenous contrast or MRI only) of a hypervascular solid liver mass with features characteristic for HCC were present in a setting of underlying risk factors, along with a clearly elevated serum alpha-fetoprotein (>100 ng/ml). A diagnosis of HCC for all patients that presented with non-AFP-producing tumors was confirmed by biopsy. Of all 127 patients, 76 (60%) were diagnosed based upon histology or cytology, while 51 (40%) were diagnosed with imaging along with other features.

### Diagnosis of cirrhosis

A diagnosis of cirrhosis was made based on a combination of clinical parameters. For patients in whom tissue (from biopsy of resection specimens) was available from the non-neoplastic liver, a diagnosis of cirrhosis was made if histologic findings of hepatic fibrosis were observed in conjunction with clinical evidence of severe hepatic dysfunction (such as hypoalbuminemia). Patients with available histologic specimens demonstrating no evidence of hepatic fibrosis were deemed non-cirrhotic. For patients in whom non-neoplastic liver tissue was unavailable, a diagnosis of cirrhosis was based upon a combination of axial imaging features (including hepatic macronodularity and ascites), serum biochemistries indicative of severe hepatic dysfunction (such as hypoalbuminemia), and physical exam findings such as documented ascites.

### Statistical analyses

Comparisons of patient cohorts were performed using Fisher’s exact test. A p value of <0.05 was used to designate a statistically significant difference among observed vs. expected outcomes.

## Results

### Demographics, age distribution, and underlying comorbidities

**Figure 1:**
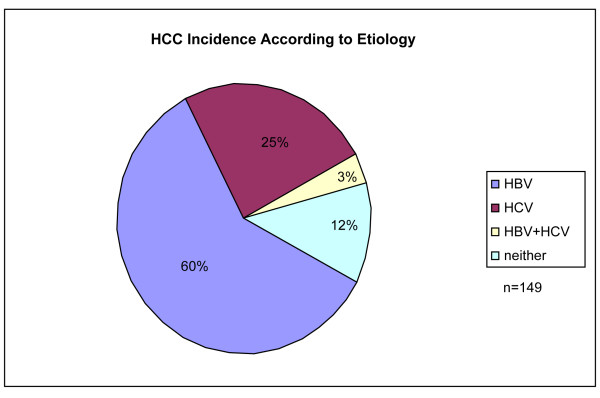
**HCC incidence according to underlying etiology (current series).** Underlying risk factors for HCC in current series closely resembles global demographic: worldwide epidemiologic data estimate HCC due to HBV in 53-54% of total cases, and due to HCV in 25%-31% of cases

A total of 149 HCC patients were enrolled during the study period: 89 (59.7%) with HBV-associated cancers, 38 (25.5%) with HCV-associated cancers, 4 (2.7%) with cancers associated with both HBV and HCV, and 18 patients (12.1%) with cancers not associated with either hepatitis virus (Figure 1). HCC patients with HBV and HCV co-infection, as well as those without underlying viral hepatitis were excluded from the present analysis. HCC developed more commonly among males in both groups, however the association to male gender was stronger in the HBV group. Only 5% of HBV-associated HCC’s developed in female patients compared with 21% of HCV-associated HCC’s (p = 0.007, Table 1). The majority of HBV-associated HCC patients were of Asian race (90%), compared with only 5% in HCV-associated HCC patients (p < 0.001, Table 1).

**Table 1:** Demographic and clinical-pathologic comparisons of patients with HCC associated with underlying HBV vs. those with HCV-associated HCC

		**All (n = 127)**	**HBV (n = 89)**	**HCV (n = 38)**	**P value †**
**Gender**					
	Male	115 (90%)	85 (95%)	30 (79%)	p = 0.007
	Female	12 (10%)	4 (5%)	8 (21%)	
**Race**					
	Asian	82 (65%)	80 (90%)	2 (5%)	p < 0.001
	Non-Asian	45 (35%)	9 (10%)	36 (95%)	
**Age**					
	Median	53	50	58	NS
	Range	21-79	21-75	47-79	
	Distribution				
	21-30	5 (4%)	5 (6%)	0 (0%)	
	31-40	18 (14%)	18 (20%)	0 (0%)	p < 0.001 ‡
	41-50	23 (18%)	21 (24%)	2 (5%)	
	51-60	50 (39%)	28 (31%)	22 (58%)	
	>60	31 (24%)	17 (19%)	14 (37%)	
**Comorbidities**					
	Mean ± S.D	1.6 ± 1.6	1.0 ± 1.1	2.9 ± 1.5	p < 0.001
**Alcohol abuse**					
		18 (15%)	6 (7%)	12 (32%)	p < 0.001
**HIV coinfection**					
		8 (6%)	1 (1%)	7 (13%)	p < 0.001
**Cirrhosis state in the non-neoplastic liver (n = 119)**					
	Non-cirrhosis	34 (29%)	34 (40%)	0 (0%)	p < 0.001
	Cirrhosis	85 (71%)	50 (60%)	35 (100%)	
**AFP levels (n = 122)**					
	Median	137	1000	37	p = 0.002
	Range	3-871,485	3-871,485	4-59,739	
	Distribution				
	≤ 9	20 (16%)	13 (15%)	7 (19%)	NS *
	9-20	10 (8%)	7 (8%)	3 (8%)	NS *
	20-100	27 (22%)	10 (12%)	17 (46%)	p < 0.001 *
	100-1,000	15 (12%)	11 (13%)	4 (11%)	p < 0.001 *
	1,000-10,000	27 (22%)	23 (27%)	4 (11%)	p = 0.01 *
	>10,000	23 (19%)	21 (25%)	2 (5%)	

† P values were obtained by Fisher’s exact test, two-tailed t test or non-parametric Mann–Whitney U test.

‡ p < 0.001 when comparing HCC developing under age 40 in HBV vs. HCV.

* p values obtained when comparing distribution with AFP cut-off at 9, 20, 100, 1,000, or 10,000 in HBV vs. HCV.

NS: not significant.

The median age upon initial diagnosis for patients with either HBV or HCV-associated HCC’s was similar (HBV, 50 years; HCV, 58 years); however, the age distribution in the two groups differed considerably. Approximately one fourth (26%) of all HBV-associated HCC’s occurred in patients aged 40 years or younger, while no cancers developed in patients under the age of 40 in the HCV group (p < 0.001, Table 1). In fact, only 2 HCC’s (5%) occurred in the HCV group at 50 years of age or less. This contrasted with the HBV group, in whom nearly half (49%) of cancers had already occurred by the age of 50 (Table 1).

As a group, patients who developed HCC associated with underlying HBV had fewer systemic comorbidities than those with underlying HCV. A mean of 1.0 underlying systemic comorbidity was present in HBV patients at the time of initial cancer diagnosis, compared with a mean of 2.9 associated systemic comorbidities in HCV patients (Table 1**,** p < 0.001). A coincident history of alcohol abuse was also less frequent in the HBV group (7% in HBV vs. 32% in HCV, p < 0.001), as was the frequency of HIV co-infection (1% in HBV vs. 13% HCV, p < 0.001).

### Co-existence of cirrhosis in the non-neoplastic liver

Patients with underlying HBV were less likely to have developed cirrhosis at the time of progression to cancer compared with patients with underlying HCV (p < 0.001, Table 1). Without exception, all patients who developed HCC in a setting of chronic HCV had established cirrhosis at the time of initial cancer diagnosis. However, 40% of patients with underlying HBV developed HCC in the absence of cirrhosis. When HCC patients with established cirrhosis due to either HBV or HCV were compared, the severity of cirrhosis as determined by the Childs-Pugh classification, was similarly distributed in both viral hepatitis groups.

### Alpha-fetoprotein production

A significant difference in serum alpha-fetoprotein (AFP)-production was observed in HCC’s associated with underlying HBV as opposed to those associated with HCV (Table 1). The median level of serum AFP in HBV-associated HCC was greater than HCV-associated HCC (1000 ng/ml vs. 37 ng/ml, p = 0.002). While the proportion of patients with normal level of serum AFP (<20 ng/ml) was similar between HBV- and HCV-associated HCC, the proportion of HCC producing higher amount of AFP (>100 ng/ml) was greater in HBV-associated HCC. Sixty five percent of HBV-associated HCC, in contrast to only 27% of HCV-associated HCC, produced AFP greater than 100 ng/ml (p < 0.001). Similarly, the proportion of HBV-associated HCC producing AFP greater than 1,000 ng/ml or 10,000 ng/ml was higher compared with HCV-associated HCC (52% vs. 16%, and 25% vs. 5%, respectively, Table 1).

### Oncologic prognostic variables

The presence upon initial cancer diagnosis of several well established oncologic variables associated with a poor cancer specific prognosis was assessed according to underlying viral hepatitis status. These include tumor size, tumor number, macrovascular invasion, and the presence of synchronous distant metastases.

Patients with HBV-associated HCC were significantly more likely to be diagnosed with larger tumors, than those with HCV-associated HCC. Seventy eight percent of HBV-associated tumors exceeded 5 cm in greatest cross-sectional diameter, as compared with only 28% of those associated with HCV (p < 0.001, Table 2). Analysis of all other selected variables failed to demonstrate a significant difference according to viral hepatitis status. Multifocal tumors and macrovascular invasion occurred with similar frequency in both the HBV and HCV groups (HBV-49% vs. HCV-54% and HBV-31% vs. HCV-24%, respectively). The incidence of synchronous distant metastases was observed with double the frequency in HBV patients, when compared to those with HCV (HBV-15% vs. HCV-6%). However, this trend did not achieve statistical significance, likely due to statistical underpowering of the study at the current sample size.

**Table 2:** Indicators of poor prognosis and their association with underlying viral hepatitis status

		**HBV**	**HCV**	**P value**
**Tumor Size** (n = 122)				
	<5 cm	19 (22%)	23 (61%)	
	>5 cm	66 (78%)	14 (28%)	p < 0.001
**Tumor Number** (n = 122)				
	solitary	43 (51%)	17 (46%)	
	multiple	42 (49%)	20 (54%)	NS
**Synchronous Metastases** (n = 116)				
	absent	71 (85%)	30 (94%)	
	present	13 (15%)	2 (6%)	NS
**Macro-Vascular Invasion** (n = 117)				
	absent	57 (69%)	26 (76%)	
	present	26 (31%)	8 (24%)	NS

### Eligibility for treatment with curative intent

Eligibility for liver transplantation and surgical resection with expected favorable long-term survival, according to underlying viral hepatitis status, was determined by application of the Milan criteria [12,13]. Patients with HBV-associated cancers were far less likely to meet these criteria upon initial HCC diagnosis than were those with HCV-associated cancers (14% vs. 34%, respectively, p < 0.05). This more commonly precluded treatment with expectation for cure in patients with HBV-associated HCC, when compared to patients with HCV.

## Discussion

Viral Hepatitis B and Hepatitis C are the most commonly implicated risk factors for HCC, with HBV responsible for the majority of cases worldwide. The epidemiologic predominance of HBV-associated HCC worldwide is largely due to the endemic nature of HBV infection in East Asian and subsaharan African populations [14]. Given the broad ethnic and racial diversity of patients seen at the single New York institution from which data was collected (Bellevue Hospital Center), patients included in the current analysis closely resemble the global demographic for HCC [2,3]. Sixty percent of all HCC cases were due to underlying HBV, and 25% were due to HCV. The majority of patients in the HBV group were of East Asian race (90%), compared to only 5% in the HCV group. This statistic potentially explains the significant disparity in age at initial cancer diagnosis among HBV vs. HCV-infected patients. HBV transmission in East Asian populations predominantly follows vertical (maternal-fetal) patterns of transmission [15]. In contrast, HBV in non-Asian populations, and HCV are more frequently transmitted in horizontal patterns as a result of exposure to infected body fluids [16-18]. Thus HCV exposure usually occurs later in life compared to HBV in the largest worldwide at risk populations. This finding from our study is consistent with previous studies in Japan showing younger detection age in HBV HCC patients compared with HCV HCC patients [19,20]. Although the mechanistic sequence of events required for progression from chronic viral hepatitis infection to HCC are poorly understood, a reasonable conclusion based on the current data could imply that the young age associated with HBV-HCC occurs as a direct result of vertical HBV transmission. Thus screening for HCC in patient populations known to be at risk for vertically-transmitted HCC should begin at an early age, no later than early adulthood.

Another important difference in the progression to HCC among HBV vs. HCV infected patients is the association to pre-existing cirrhosis at the time of initial cancer diagnosis. The occurrence of HCC in the absence of cirrhosis in a substantial percentage of HBV-HCC patients (40%), compared to the invariable association to established cirrhosis in those with HCV-HCC, suggests a potential cirrhosis-independent pathway to cancer unique (among viral hepatitis-associated cancers) to HBV-HCC. Much of the literature suggests a generally accepted common pathway to hepatocarcinogenesis, proposed in both HBV and HCV patients, based on: active viral replication, chronic hepatocellular necroinflammatory activity, hepatic parenchymal fibrosis, cirrhosis, and eventually cancer [21-23]. Although this model appears valid for patients with HCV, some with HBV do not experience such a predictable sequence of events prior to developing liver cancer [24-26].

Considerations relevant to the care of patients who are subject to a cirrhosis-independent pathway to HCC should lead to proposed changes in current widely-adopted practices of screening for HCC in patients with chronic HBV [27-29]. Proposed changes in surveillance practices, particularly in those suspected of perinatal infection, should include a high level of vigilance for cancer even in the absence of cirrhosis. Several levels of evidence, in addition to the current data, reinforce that HBV patients are at risk for HCC even prior to the development of cirrhosis. Thus HCC screening should not be withheld until evidence of active hepatocellular necroinflammation or cirrhosis are observed.

Several additional significant clinical-pathologic differences in HCC were also observed according to underlying viral hepatitis status, including AFP production. This observation is particularly interesting, given that the utility of serum AFP as a cancer screening tool has been the subject of considerable recent debate, leading some experts in the field to not advocate its use as a tumor marker [30]. In the current study, median AFP level in HCV-associated HCC was 37 ng/ml, compared with 1000 ng/ml in HBV-associated HCC, and only 27% among HCV-associated cancers produced serum AFP greater than 100 ng/ml, compared with 65% in of HBV-associated HCC. This result suggests a higher sensitivity of serum AFP as a tumor marker when used in patients with HBV, but maybe less so in screening patients with HCV for cancer.

Oncologic variables with established prognostic significance were evaluated according to viral hepatitis status. Both macrovascular invasion and synchronous metastases occurred with greater frequency in the HBV-HCC group, although these observations did not achieve statistical significance. Only tumor size on initial HCC diagnosis, an important prognostic indicator, was statistically different among HBV vs. HCV patients [31-33]. This tumor size discrepancy may be explainable by less rigorous cancer screening recommendations for patients with chronic HBV, compared to more uniform and frequent screening programs for patients with HCV [34,35]. Many of the accrued HBV-HCC patients would not have met current American Association for the Study of Liver Diseases (AASLD) guidelines for HCC screening due to their young age, absence of cirrhosis or other variables [30]. Thus a relatively delayed cancer diagnosis may be attributable in part to under screening of HBV patients for HCC, when compared to those with chronic HCV.

Often due to initial presentation with large tumors, patients in the HBV-HCC group were less likely to meet Milan eligibility criteria for liver transplantation (single tumor <5 cm in greatest diameter or multiple tumor each <3 cm in greatest diameter) [12]. Due to the similar prognostic importance of applying Milan criteria to surgical resection series, this association to larger tumors also predicted a lower likelihood of surgical resection with expected favorable long term survival [13,36,37]. Overall a staggeringly small fraction of all patients with HCC associated with either hepatitis virus met Milan criteria, underscoring the fact that most patients (80%) cannot be treated with the two most effective treatments or an expectation for favorable long term outcome. However, despite this observation in both viral hepatitis patients, inoperable or poor prognosis cancers were significantly more common in patients with HBV. Whether HBV or HCV viral etiology is an independent prognostic factor in patients with HCC following surgical resection or liver transplantation remains controversial and requires further investigation [38-42].

## Conclusions

In summary, patients with HBV-associated HCC were more likely to present with poor-prognosis cancers, often at a young age, and in the absence of cirrhosis. In contrast, patients with HCV-associated HCC often were diagnosed in a setting of pre-existing cirrhosis, with multiple comorbidities, but with more favorable oncologic features. These data suggest a role for more aggressive screening and management of chronic HBV patients, particularly those subjected to maternal-fetal viral transmission. The importance of earlier screening and more aggressive treatment is especially emphasized by the advanced oncologic nature of HCC associated with HBV on typical initial diagnosis.

## Abbreviations

HCC, Hepatocellular carcinoma; HBV, Hepatitis B; cccDNA, Covalently closed circular DNA; AFP, Alpha-fetoprotein; AASLD, American Association for the Study of Liver Diseases.

## Competing interests

The authors declare that they have no competing interests.

## Authors’ contributions

SPH conceived of, designed and supervised the study and drafted the manuscript. NNR participated in data acquisition, GAV participated in data acquisition, EK participated in data acquisition, WL participated in data entry and acquisition, QW participated in data analysis and manuscript writing, HTY performed histological analysis. All authors read and approved the final manuscript.

## Pre-publication history

The pre-publication history for this paper can be accessed here:

http://www.biomedcentral.com/1471-230X/12/64/prepub
